# Rescue of Misfolded Organic Cation Transporter 3 Variants

**DOI:** 10.3390/cells12010039

**Published:** 2022-12-22

**Authors:** Thomas J. F. Angenoorth, Julian Maier, Stevan Stankovic, Shreyas Bhat, Sonja Sucic, Michael Freissmuth, Harald H. Sitte, Jae-Won Yang

**Affiliations:** 1 Institute of Pharmacology, Center for Physiology and Pharmacology, Medical University of Vienna, Währingerstraße 13A, 1090 Vienna, Austria; 2 Department of Physics, Université de Montréal, 1375 Avenue Thérèse-Lavoie-Roux, Montréal, QC H3T 1J4, Canada; 3 Department of Pharmacology and Physiology, Université de Montréal, 2960 Chemin de la Tour, Montréal, QC H3T 1J4, Canada

**Keywords:** 4-PBA, µ-pifithrin, 17-DMAG, progesterone, corticosterone, pharmacochaperoning, chemical chaperone

## Abstract

Organic cation transporters (OCTs) are membrane proteins that take up monoamines, cationic drugs and xenobiotics. We previously reported novel missense mutations of organic cation transporter 3 (OCT3, SLC22A3), some with drastically impacted transport capabilities compared to wildtype. For some variants, this was due to ER retention and subsequent degradation of the misfolded transporter. For other transporter families, it was previously shown that treatment of misfolded variants with pharmacological and chemical chaperones could restore transport function to a certain degree. To investigate two potentially ER-bound, misfolded variants (D340G and R348W), we employed confocal and biochemical analyses. In addition, radiotracer uptake assays were conducted to assess whether pre-treatment with chaperones could restore transporter function. We show that pre-treatment of cells with the chemical chaperone 4-PBA (4-phenyl butyric acid) leads to increased membrane expression of misfolded variants and is associated with increased transport capacity of D340G (8-fold) and R348W (1.5 times) compared to untreated variants. We herein present proof of principle that folding-deficient SLC22 transporter variants, in particular those of OCT3, are amenable to rescue by chaperones. These findings need to be extended to other SLC22 members with corroborated disease associations.

## 1. Introduction

Organic cation transporters (OCTs) 1–3 (OCT1-3, SLC22A1-3) are facilitative, poly-specific transporters. Their eponymous action is to mediate the cellular uptake of endogenous—e.g., monoamines—and exogenous cationic molecules, i.e., drugs and xenobiotics [1]. As low-affinity, high-capacity monoamine transporters, OCTs, and particularly OCT3, complement the action of the high-affinity, low-capacity SLC6 neurotransmitter-sodium symporters (NSS) and are thus essential for maintaining monoaminergic equilibrium [2,3]. Due to their interaction with a plethora of cationic medical drugs, they also define the pharmacokinetics of clinically relevant drugs, and they shape drug–drug interactions [4,5,6].

OCT1 and OCT2 are most abundantly present in hepatocytes and the tubular epithelial cells of the kidney, respectively. In contrast, OCT3 is more widely expressed, namely in the heart, central nervous system (CNS), liver, placenta, muscle tissue, intestinal tract and adipocytes [1]. OCT3 expression in cardiomyocytes is essential to support cardiac contractility by mediating uptake of norepinephrine and its subsequent binding to intracellular β1-adrenergic receptors [7]. Similarly, OCT3-expressing adipocytes participate in clearance of norepinephrine [8]. Cardiac OCT3 has been implicated in doxorubicin-related cardiotoxicity through uptake of the drug [9]. OCT3 also mediates the cellular uptake of platinum-based compounds; thus, the expression of OCT3 determines—at least in part—the susceptibility of cancer cells to their cytotoxic action [10,11]. OCT3 is widely expressed in the CNS, in particular in neurons and glial cells of brain regions that contain monoaminergic projections [12,13]. While its exact role in various neuropsychiatric diseases has not yet been fully illuminated, it is speculated that disruption of CNS-located OCTs impacts maintenance of monoaminergic equilibrium and neurotransmission [3,14,15,16].

With very few exceptions, intrinsic membrane proteins are synthesized in the endoplasmic reticulum (ER), where their folding is assisted by both luminal ER-resident and cytosolic chaperones [17,18]. The folding trajectory is monitored by a quality control system: misfolded proteins are removed by ERAD (ER-associated degradation), a dedicated machinery which feeds the dislocated proteins into the proteasome [17,19].

In the solute carrier family of membrane transporters, the list of point mutations that are linked to diseases is rapidly expanding; the majority of these mutations are thought to result in a folding defect of the encoded mutant protein [20]. Folding of most solute carriers including SLC22 family members is poorly understood. In contrast, the folding trajectory of the monoamine transporters of the SLC6 family, in particular of the serotonin transporter (SERT, SLC6A4), has been studied in considerable detail [21,22,23,24].

Chemical and pharmacological chaperones (pharmacochaperones) can promote protein folding and rescue folding-deficient, disease-related variants of the dopamine transporter (DAT, SLC6A2) [25,26,27,28,29,30,31,32], of the creatine transporter (CrT1, SLC6A8) [25] and of glycine transporter-2 (GlyT2, SLC6A5) [33]. Likewise, inhibitors of endogenous (i.e., proteinaceous) chaperones can relieve stringent quality control and thus restore surface expression [34]. This concept has been preclinically and clinically employed for a variety of targets and diseases. Some examples include tafamidis for wildtype tranthyretin amyloidosis (ATTR), ivacaftor and derivatives for treatment of certain cystic fibrosis cases (specific mutations in the CFTR gene) and chaperones targeting vasopressin type 2 receptor mutations causing nephrogenic diabetes insipidus, among others [17,35,36,37]. We recently reported the structure of OCT3 and characterized 24 novel genetic variants in vitro [38]. We noticed two variants (OCT3-D340G and -R348W), which had greatly reduced transport activity because of decreased cell surface expression. Here we explored whether these OCT3 mutants were retained in the ER because of misfolding. Our experiments provide a proof of principle for pharmacological rescue of OCT3 transporter function and thus extend pharmacochaperoning to the SLC22 family.

## 2. Materials and Methods

### 2.1. Materials and Chemicals

[^3^H]MPP^+^ (N-methyl-4-phenylpyridinium, 82.9 Ci/mmol) and ULTIMA Gold scintillation mixture were purchased from PerkinElmer Life Science (Waltham, MA, USA). Decynium-22 was ordered from Synthon Chemicals (Bitterfeld, Wolfen, Germany). All other chemicals, including 4-phenyl butyric acid, and cell culture media were purchased from Sigma-Aldrich (St. Louis, MO, USA) and Sarstedt (Nuembrecht, Germany).

### 2.2. Cell Culture

hOCT3 was C-terminally YFP-tagged and single point mutations were introduced as previously described [38]. Constructs were verified by sequencing (LGC Genomics, Berlin, Germany). In brief, using jetPRIME© (Polyplus Transfection; VWR International GmbH, Vienna, Austria) reagent (for a 10 cm dish with 1–2 × 10^6^ cells in 10 mL serum containing medium at 60–80% confluency: 500 µL jetPRIME^©^ buffer, 10 µg DNA and 20 µL jetPRIME^©^ reagent), HEK293 cells were transfected with the respective plasmid containing the wildtype or variant, according to the manufacturer’s instructions (VWR International GmbH, Vienna, Austria) [39]. After selection pressure was maintained for 14 days by adding 100 µL geneticin (G418, 50 mg·mL^−1^), 500,000 cells were FACS-sorted (fluorescence-activated cell sorting) according to expression levels to establish polyclonal cell lines. In cell culture, cells were maintained at high glucose (4.5 g·L^−1^), l-glutamine-containing (584 mg·L^−1^) Dulbecco’s Modified Eagle Medium (Sigma-Aldrich, St. Louis, MO, USA) with 10% heat-inactivated foetal bovine serum (FBS, Sigma-Aldrich), penicillin (1 U·mL^−1^, Sigma-Aldrich) and streptomycin (1 µg·mL^−1^, Sigma-Aldrich). To maintain selection pressure the medium was supplemented with 50 µg·mL^−1^ geneticin (G418). Cells were maintained in 10 cm cell culture dishes (Greiner) at 37 °C and 5% CO_2_ in a humidified incubator.

### 2.3. Radiotracer Uptake Assays

For uptake and single-point uptake assays, HEK293 cells expressing the wildtype or mutant YFP-tagged hOCT3 transporter were seeded on poly-D-lysine coated 96-well plates at a density of approx. 60,000 cells per well with and without treatment. After 24 h, the medium was replaced with 200 µL Krebs-Hepes buffer (10 mM HEPES NaOH, pH 7.3, 120 mM NaCl, 3 mM KCl, 2 mM CaCl_2_ and 2 mM glucose) and subsequently incubated for 10 min in the presence of 0.05 µM [^3^H]MPP^+^. For full uptake curves, untritiated MPP^+^ was added in increasing concentrations, as previously described in detail [35]. For nonspecific uptake, the cells were pre-treated in the presence of 200 µM decynium-22. After 10 min, the reaction was terminated by aspiration and subsequently washed with 200 µL ice-cold Krebs-Hepes buffer. The cells were then treated with 200 µL of scintillation cocktail to release the retained radioactivity, which was quantified with a beta-scintillation counter. To assess cell loss in the washing steps, six wells per plate were washed three times with Krebs-Hepes buffer and counted in a Neubauer chamber.

### 2.4. Confocal Microscopy

Images were taken with a Nikon A1R + laser scanning confocal microscope system with a 60× NA 1.4 oil immersion objective (Nikon, Vienna, Austria). After aspiration of cell culture medium, cells were incubated with trypan blue (0.4%, Sigma-Aldrich) for 10 min and subsequently washed with KHB. eYFP fluorescence was excited with a 488 nm laser line, trypan blue with a 561 nm laser and monomeric BFP with a 399 nm laser. A 525/50 nm, 595/50 nm or 454/50 nm emission filter was used for eYFP, trypan blue or mTagBFP, respectively. Images were analysed as previously described [38]. In brief, images, taken on at least three separate days, were analysed with Fiji ImageJ 1.53c. Membrane expression was assessed by drawing the cell membrane and the interior of the call and calculating relative expression at the membrane.

### 2.5. Immunoblotting (IB)

Cells stably expressing YFP-tagged OCT3 proteins were solubilized in lysis buffer containing 1% Triton X-100, 20 mM Tris-HCl (pH 8.0), 150 mM NaCl, 1 mM EDTA, 1 mM sodium orthovanadate, 5 mM sodium fluoride, 5 mM sodium pyrophosphate and a protease inhibitor cocktail (Roche, Mannheim, Germany). Proteins were size-fractionated on SDS-PAGE gels and transferred to polyvinylidene fluoride membranes (Waters, Milford, MA, USA) for immunostaining with anti-GFP antibody (Invitrogen, Waltham, MA, USA).

### 2.6. Data and Statistical Analysis

Vmax and Km values were calculated, data analysed, and graphs plotted using GraphPad Prism 9.2.0 (GraphPad Software Inc., San Diego, CA, USA). All data stem from at least three independent experiments (*n* = 3) executed in triplicates and are shown as mean ± SD.

## 3. Results

**Figure 1:**
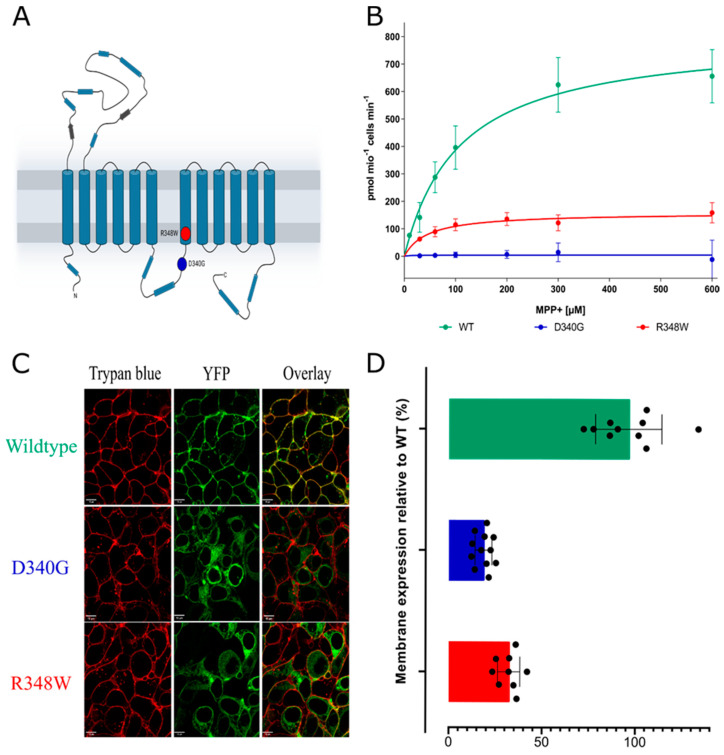
(**A**) Snake plot of the structure of OCT3 with the D340G and R348W variants labelled. (**B**) Uptake assays of wildtype OCT3 and the two variants, OCT3-D340G (D340G) and OCT3-R348W (data are mean ± standard deviation from three independent determinations in triplicate). (**C**) Representative confocal images of the wildtype and variants. The cell membrane was visualized by staining with trypan blue. (**D**) Quantitative analysis of membrane localization, assessed on at least 10 cells, measured on three separate days.

OCT3 harbours the major facilitator superfamily fold with 12 transmembrane (TM) segments organized in a 6 plus 6 internal symmetry and a prominent extracellular domain (ECD). The mutations D340G and R348W are located in the large intracellular loop-3 (ICL3) and in TM7, which is itself in the vicinity of the ICL3, respectively (see Figure 1A). We used the tritiated OCT substrate 1-methyl-4-phenylpyridinium ([^3^H]MPP^+^) to determine the activity of OCT3-D340G and OCT3-R348W after heterologous expression in HEK293 cells (Figure 1B): cellular uptake mediated by OCT-R348W was approximately tenfold lower than that of wildtype OCT3 (Table 1). The reduction in transport velocity was even more pronounced for OCT-D340G, where residual Vmax was only about 2% of the Vmax of wildtype OCT3.

We examined the cellular distribution of YFP-tagged wildtype and mutated OCT3 by confocal microscopy: the fluorescence of wildtype OCT3 was predominantly colocalized with trypan blue, which was used to delineate the cell surface (top row in Figure 1C). In contrast, OCT-D340G was confined to the intracellular compartment (middle row in Figure 1C). Similarly, the bulk of OCT3-R348W was visualized within the cells and only very modest amounts colocalized with trypan blue (bottom row in Figure 1C). For quantitative image analyses confirming these findings, see Figure 1D. The observations summarized in Figure 1 suggested that the two mutant OCT3 variants were retained in the ER because they were folding-deficient. We verified this conjecture by first examining colocalization of the proteins with calnexin.

**Figure 2:**
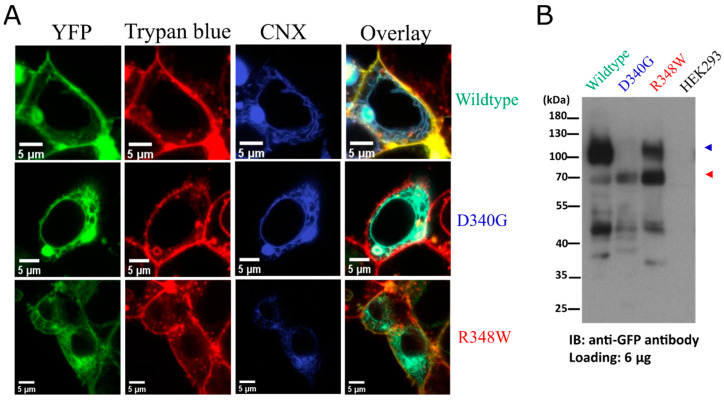
(**A**) Representative images of YFP-tagged wildtype OCT3 (top row), OCT3-D340G (middle row) and OCT3-R348W (bottom row) were captured by confocal microscopy to visualize the YFP-tagged transporter (left hand column), trypan blue (second column) and mTagBFP-tagged calnexin (CNX; third column). The overlay is shown in the right-hand column. (**B**) Western blot of lysates (6 µg) prepared from HEK293 cells expressing YFP-tagged wildtype OCT3, OCT3-D340G, OCT-R348W and from untransfected HEK293 cells. The red and the blue arrow indicate the position of the ER-resident core-glycosylated transporter and mature glycosylated transporter, respectively.

HEK293 cells were co-transfected with plasmids encoding the individual YFP-tagged OCT3 variant and fluorescently tagged calnexin (mBFP-CNX) as a marker of the ER membrane [40]. Representative images, which were captured by confocal microscopy, are shown in Figure 2A: it is evident that wildtype OCT3 did not colocalize with calnexin but rather with the membrane dye trypan blue (top row in Figure 2A). In contrast, OCT3-D340G almost exclusively colocalized with calnexin (middle row in Figure 2A). Consistent with the data shown in Figure 1C, there was a modest level of OCT-R348W at the cell surface, but the bulk of the protein colocalized with calnexin (bottom row in Figure 2A). Membrane proteins are subject to N-linked core glycosylation in the endoplasmic reticulum. After delivery to the Golgi apparatus, additional sugar moieties are attached, and the protein thus acquires its mature glycan structure. OCT3 harbours three consensus sites for N-linked glycosylation in the ECD (N72, N99 and N119). Core glycosylated proteins are smaller in apparent molecular weight and thus migrate faster than the species that harbour the mature glycan moieties. Accordingly, we electrophoretically resolved the YFP-tagged transporter proteins and visualized their distribution by immunoblotting: wildtype YFP-tagged OCT3 migrated predominantly as a broad band at 100 to 120 kDa and only a minor fraction was seen in the 70 kDa range (left-hand lane in Figure 2B). Consistent with the observation that OCT3-D340G was confined to the ER, we only detected immunoreactivity in the 70 kDa range, corresponding to the core glycosylated protein (second lane in Figure 2B). Similarly, the bulk of OCT3- R348W immunoreactivity was in the 70 kDa range, but there were appreciable amounts in the 100 to 120 kDa range (third lane in Figure 2B). This indicates that a fraction of OCT3-R348W was exported from the ER and did reach the Golgi apparatus. We also note that there was immunoreactivity in the molecular mass range below 55 kDa; these likely reflect degradation products rather than non-specific staining because these bands were absent in lysates prepared from untransfected HEK293 cells (right-hand lane in Figure 2B).

**Figure 3:**
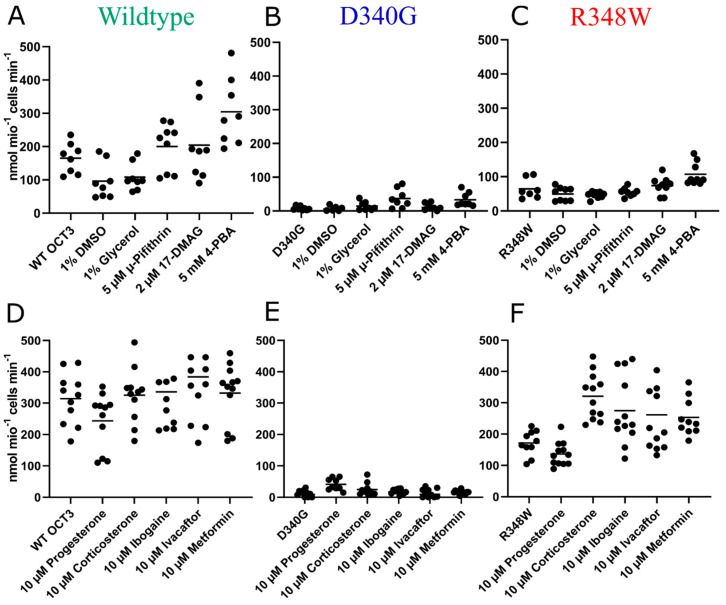
Single-point uptakes of [^3^H]-MPP^+^ by the wildtype (**A**,**D**) and variants D340G (**B**,**E**) and R348W (**C**,**F**) exposed to a variety of compounds. Experiments were conducted in triplicate, 3–4 times. Kruskal–Wallis tests with Dunn’s correction for multiple testing were employed, comparing the untreated transporter variant with all treatments.

**Figure 4:**
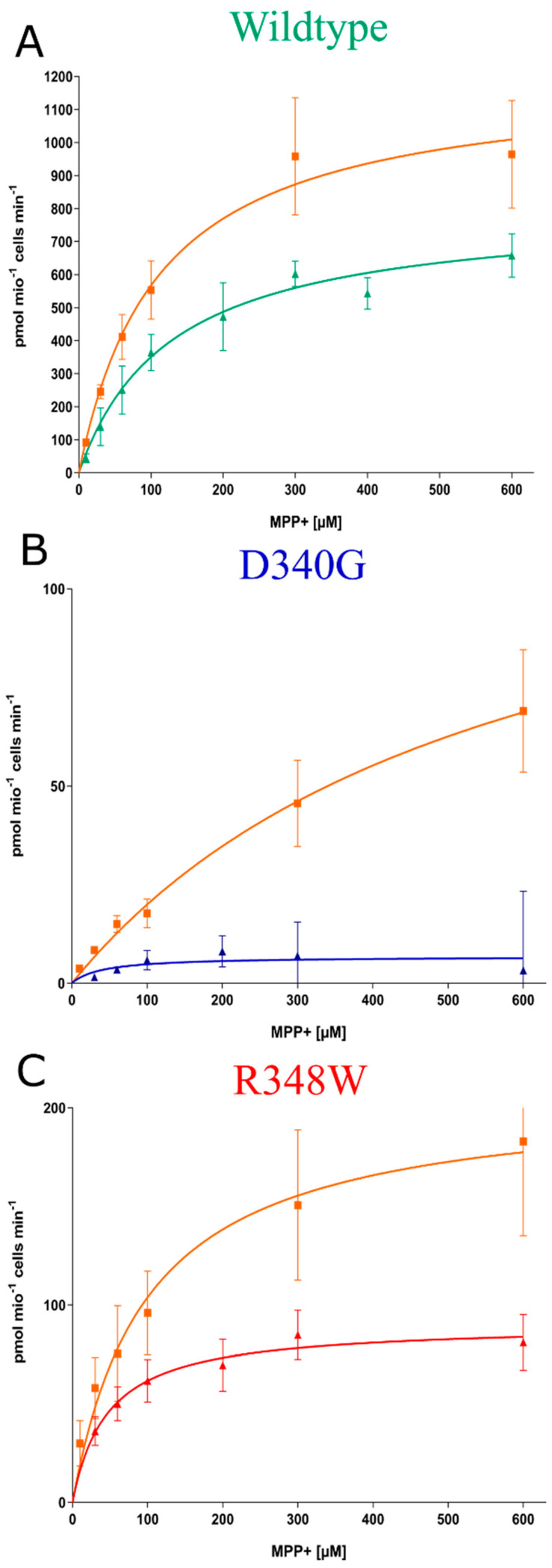
(**A**–**C**) Radiotracer uptake assays of [^3^H]-MPP^+^ taken up by the wildtype and variants with 4-PBA pre-treatment (orange line) and without. *n* = 3–4, in triplicate. Data are given as mean ± standard deviation.

These observations suggested that the ER export of OCT3-D340G and of OCT3-R348W was impaired because they were misfolded. If this was the case, their folding and subsequent surface expression ought to be restored by chemical and/or pharmacological chaperones. Substrate uptake is contingent on surface delivery of the transporter and thus allows for monitoring rescue of misfolded transporters by chemical or pharmacological chaperoning. Accordingly, we preincubated HEK293 cells expressing wildtype or mutated OCT3 for 24 h with chemical chaperones, i.e., DMSO (dimethyl sulfoxide), glycerol and 4-PBA (4-phenylbutyrate), and heat-shock protein (HSP) inhibitors pifithrin-μ and 17-DMAG (17-desmethoxy-17-N,N-dimethylaminoethylamino-geldanamycin), which target HSP70 and HSP90, respectively. Thereafter, substrate uptake was measured. Neither preincubation with DMSO nor with glycerol increased [^3^H]MPP^+^ uptake mediated by wildtype OCT3 or by the mutated OCT3 variants (Figure 3A–C). Pifithrin-μ and 17-DMAG pre-treatment led to inconsistent increases across variants, but the effect in the WT and mutants was most marked and consistent after 4-PBA treatment (see ibid). 4-PBA treatment led to a fifty percent increase in MPP^+^ transport capabilities in the wildtype and R348W variant, while uptake of the D340G mutant was on average 8-fold higher. Statistical analyses comparing untreated and treated conditions for each variant revealed this difference to be significant for the D340G variant (adjusted *p*-value: 0.012) and bordering significance for R348W (adjusted *p*-value: 0.055). In addition, treatment with pifithrin-μ led to a significant increase in uptake in variant D340G (adjusted *p*-value: 0.011). To see whether we could extend these findings to pharmacochaperones, we pre-treated the wildtype and variants with the steroid hormones progesterone and corticosterone, inhibitors of OCT3; ibogaine, which has been shown to enhance serotonin and dopamine transporter expression [41]; ivacaftor, a pharmacochaperone used in the treatment of cystic fibrosis [35]; and metformin, an antidiabetic biguanide and substrate of OCT3 (Figure 3D–F). Pre-treatment with progesterone led to significantly increased uptake in variant D340G (adjusted *p*-value: 0.0009) and corticosterone pre-treatment had positive effects on R384W (adjusted *p*-value: 0.0007). Due to the pronounced effects of 4-PBA treatment on variants in single-point uptakes, we conducted further uptake experiments, deriving full curves, offering more kinetic information. 4-PBA pre-treatment increased the Vmax of D340G approximately sevenfold and twofold for variant R384W (see Figure 4A–C, Table 1).

## 4. Discussion

Polymorphisms are widespread in enzymes and transporters, which determine the disposition of drugs and xenobiotics. This is also true for OCT1 [42] and OCT2 [43]. In contrast, currently there is little evidence for OCT3 polymorphism in human populations, but more than 300 infrequently occurring missense coding variants have been reported so far [38,44]. Their significance is poorly understood. In the present study, we designed experiments to confirm that OCT3-R348W and OCT3-D340G were hypomorphic and loss-of-function alleles, respectively: their pronounced reduction and loss of transport activity, respectively, were accounted for by ER retention. This conclusion is based on two independent lines of evidence: (i) confocal microscopy visualized the bulk of the mutated proteins colocalized with calnexin; (ii) their electrophoretic mobility showed that they predominantly or exclusively accumulated as core-glycosylated—i.e., ER-resident—species. Consistent with virtual absence at the cell surface, OCT3-D340G failed to acquire any detectable mature glycan. Similarly, the mature glycosylated species of OCT3-R348W was substantially lower than that of wildtype OCT3. Finally, the transport activity of OCT3-R348W and OCT3-D340G was restored—in part—by chemical and pharmacological chaperoning. Thus, taken together, these observations are consistent with the interpretation that OCT3-R348W and OCT3-D340G are folding-deficient variants.

Pharmacochaperones must be lipophilic to permeate the cell membrane and reach the folding intermediates in the ER. Steroid hormones are lipophilic; several steroid hormones are known to inhibit OCT3 with µM affinities [45]. We selected two compounds, i.e., corticosterone and progesterone, to explore their pharmacochaperoning action. Corticosterone was effective in rescuing OCT3-R348W. In contrast, cellular preincubation with progesterone restored uptake mediated by OCT3-D340G, but it failed to enhance transport by OCT3-R348W. This differential response is not without precedent: only a fraction of disease-associated folding-deficient DAT mutants are rescued by noribogaine [28] and/or bupropion [30]. Subtle structural variations in DAT ligands can greatly enhance their pharmacochaperoning efficacy and expand the spectrum of DAT mutants in which folding can be restored [29,41]. Four mechanisms have been proposed to underlie the action of pharmacochaperones: (i) binding to and stabilization of the folded state; (ii) binding to and stabilization of folding intermediates; (iii) inhibition of aggregate formation; and (iv) dissolution of aggregates [46]. The first two of these mechanisms are the most plausible to account for the rescue of misfolded transporters by pharmacochaperones and circumstantial evidence indicates that binding to and stabilization of folding intermediates is important [20]. It was somewhat surprising that progesterone failed to rescue OCT3-R348W, because the folding deficiency of this mutant was less pronounced than that of OCT3-D340G. At the very least, this observation indicates that OCT3-R348W and OCT3-D340G are stalled at different positions in the folding trajectory. This conclusion is further supported by the observation that the two mutants differed in their susceptibility to correction by HSP inhibition: the HSP70 inhibitor pifithrin-µ seemed more effective in partially rescuing OCT3-D340G. There is again precedent for this observation: folding-deficient SERT mutants differ widely in their susceptibility to correction by HSP inhibition, even if the disruptive point mutations are in close vicinity [21]. The same is true for disease-associated, folding-deficient mutants of CRT1 [25].

Cellular preincubation with the chemical chaperone 4-PBA also increased the transport velocity of wildtype OCT3. The mechanism underlying the chaperoning action of 4-PBA is not clear [47], but it affects the balance of expression of HSP, which assists folding and directs folding intermediates to degradation [48,49]. Degradation products were detected by immunoblotting for both wildtype and mutant OCT3. Taken together, these findings indicate that a fraction of wildtype OCT3 also incurs a folding problem, which is aggravated by point mutations. This conclusion is also supported by the finding that relieving quality control by inhibition of HSP also seemingly enhanced the transport velocity of wildtype OCT3. Inhibition of HSP90 has also been previously found to increase surface levels of endogenously expressed SERT [21]. During substrate translocation, transporters visit several distinct conformations. Point mutations, which impede folding, can also interfere with this conformational cycle [28]. Cellular preincubation with 4-PBA enhanced transport in all three variants that were investigated, i.e., wildtype OCT3, OCT3-R348W and OCT3-D340G. The apparent affinity of wildtype OCT3 and OCT3-R348W for substrate was not affected by 4-PBA. In contrast, even when rescued, the apparent MPP^+^ affinity was reduced in OCT3-D340G. The D340G mutation is in the large third intracellular loop-3 and thus far removed from the substrate binding site of OCT3 [38]. While indirect effects on substrate binding cannot be formally ruled out, it is more likely that the D340G mutation impairs the conformational cycle.

The constitutive knockout of OCT3 only causes subtle effects in mice; in fact, contradictory conclusions, i.e., increased and decreased levels of anxiety, were reached if these mice were analysed in behavioural paradigms [12,50]. Similarly, people harbouring hypomorphic or loss-of-function alleles of OCT3 do not show any obvious phenotype; if anything, loss of function protects against neuropsychiatric disorder [38]. A possible explanation for the lack of overt phenotype is compensation for the loss of OCT3 during neuronal development. Misfolded variants, which can be effectively rescued by pharmacochaperoning, provide an alternative approach to interrogate the role of OCT3, because pharmacochaperoning allows for reversible cell surface expression of the transporter. OCT expression in tumour cells was shown to be essential for the uptake of cationic chemotherapeutics [51,52,53,54]. Increased tumour expression of OCT2 and OCT3 was associated with increased progression-free survival in colorectal cancer patients treated with platin derivatives [10,54,55]. Therefore, inducing higher tumour OCT expression by adjuvant therapy with a chaperone might represent an attractive future anti-cancer strategy to be explored in an in vivo model.

Extrapolation of our results to OCT1, OCT2 and other SLC22 transporters warrants further investigation in the future. Usefulness might be high due to OCT1 and OCT2 shaping pharmacokinetics and being highly polymorphic transporters [56]. In particular, four polymorphisms of OCT1 (R61C, G401S, M420del and G465R) occur very frequently (up to 18 percent) and are associated with vastly decreased transport capabilities [57,58]. Polymorphisms were found to alter the pharmacokinetics of metformin [59,60]. Similarly, various variants of OCT2 have been found, with S270A being the most frequent [43]. Associated with decreased uptake, carriers of the variant show altered pharmacokinetics of metformin and cisplatin [61,62]. Additional potential targets for chaperoning attempts in the SLC22 family are mutated OCTN2 transporters (SLC22A5; organic cation/carnitine transporter), causing primary systemic carnitine deficiency (CDSP) [63,64,65]. CDSP is inherited autosomal recessively and tested for in new-born screenings [66]. Idiopathic renal hypouricemia can be caused by a mutation of URAT1 (SLC22A12; organic anion/urate transporter) [67]. Hypouricemia can be regarded as mostly beneficial but can lead to nephrolithiasis and acute kidney failure, therefore rendering URAT1 another potential target to test pre-clinically [64,67].

## 5. Conclusions

We provide a proof of principle that folding-deficient OCT3 variants are amenable to rescue by chaperones. These findings may be extended to other SLC22 members with corroborated disease associations. In addition, 4-PBA as adjuvant therapy in OCT3-expressing tumour cells might be of potential clinical benefit and warrants in vivo testing.

## Figures and Tables

**Table 1:** Km and Vmax values of the wildtype and variants in untreated and 4-PBA pre-treated conditions. Values are given with 95% confidence intervals.

Transporter Variant	Untreated		4-PBA Pre-Treated	
	K_m_ (µM)	V_max_ (pmol/10^6^ cells/min)	K_m_ (µM)	V_max_ (pmol/10^6^ cells/min)
WT	127.9 (100.7–162.7)	798.6 (733.8–873.3)	110.8 (84.79–136.8)	1196 (1096–1296)
D340G	249.6 (0.00–1405)	21.54 (−24.58–67.65)	574.0 (327.1–820.9)	134.4 (100.3–168.6)
R348W	46.68 (15.0–112.3)	90.39 (71.41–116.1)	99.06 (66.01–132.1)	206.8 (182.9–230.7)

## Data Availability

The data presented in this study are available from the corresponding author upon reasonable request.
